# High yield of cardiac MRI in the investigation of elderly heart failure patients of African descent

**DOI:** 10.1186/1532-429X-15-S1-P166

**Published:** 2013-01-30

**Authors:** Jason Dungu, Joseph Marshall, Mark S O'Donnell, Brendan Madden, Carol J Whelan, Philip N Hawkins, Lisa Anderson

**Affiliations:** 1 St George's University of London, London, UK; 2 UK National Amyloidosis Centre, London, UK

## Background

Systematic review of 1142 patients with acute heart failure presenting to St George's Hospital in South London has revealed a high prevalence of transthyretin (TTR) amyloidosis in patients of African descent. This is in keeping with the reported gene frequency of variant TTR V122I in 4% of African Americans. Availability of on-site cardiac MRI (CMR) has facilitated this high detection rate.

## Methods

A total of 104 Afro-Caribbean patients aged over 65 years presented with acute heart failure between 2005 and 2011. In 39 patients heart failure was attributed to ischaemic (n = 14), valvular (n = 9) or dilated cardiomyopathy (n = 16), and 18 patients had contraindication to CMR scanning. Of the remaining 47 patients, 39 attended for CMR (83%). CMR was performed using a 1.5T GE Signa HDxt scanner prior to 2010 and a 3.0T Philips Achieva scanner since 2010. Cardiac biopsy (n = 17) was performed in cases of suspected amyloidosis.

## Results

Mean age was 75 ± 6 years (range 65-88) with a male predominance (72%). Cardiac amyloidosis was confirmed in 15 patients (38.5%) following suggestive CMR, including 12 patients (31%) with variant ATTR V122I, 2 with senile systemic amyloidosis and one with AL amyloidosis. A further patient with characteristic CMR for cardiac amyloidosis died before further investigations could be completed. Other diagnoses were as follows: dilated cardiomyopathy in 8 patients (20.5%); hypertensive cardiomyopathy in 8 patients (20.5%); ischaemic cardiomyopathy in 4 patients (10.3%); and hypertrophic cardiomyopathy in 3 patients (7.7%). Two scans were falsely reported with a differential diagnosis of cardiac amyloidosis. Both patients had a long history of hypertension and diffuse late gadolinium enhancement on CMR; subsequent cardiac histology demonstrated extensive myocardial fibrosis only and both were classified as hypertensive cardiomyopathy.

## Conclusions

Cardiac MRI scanning in the elderly Afro-Caribbean heart failure population yields a high rate of infiltrative cardiomyopathy and should be considered in all patients in whom heart failure cannot be attributed to ischaemic or valvular heart disease. Cardiac amyloidosis due to variant transthyretin V122I was diagnosed in one-third of all Afro-Caribbean patients over 65 years following cardiac MRI in our centre.

## Funding

J.D. is supported by a British Heart Foundation Clinical Research Training Fellowship grant no. FS/09/063/28026

